# Study of Inkjet-Printed Silver Films Based on Nanoparticles and Metal-Organic Decomposition Inks with Different Curing Methods

**DOI:** 10.3390/mi11070677

**Published:** 2020-07-12

**Authors:** Peng Xiao, Yicong Zhou, Liao Gan, Zhipeng Pan, Jianwen Chen, Dongxiang Luo, Rihui Yao, Jianqiu Chen, Hongfu Liang, Honglong Ning

**Affiliations:** 1 School of Physics and Optoelectronic Engineering, Foshan University, Foshan 528000, China; xiaopeng@fosu.edu.cn (P.X.); iamjwen@126.com (J.C.); 2 Institute of Polymer Optoelectronic Materials and Devices, State Key Laboratory of Luminescent Materials and Devices, South China University of Technology, Guangzhou 510640, China; zhou.yicong@mail.scut.edu.cn (Y.Z.); c.jianqiu@mail.scut.edu.cn (J.C.); 201530291429@mail.scut.edu.cn (H.L.); ninghl@scut.edu.cn (H.N.); 3 Air Force Representative Office in Zunyi District, Zunyi 563000, China; 15985282924@139.com; 4 Guizhou Meiling Power Supply Co., Ltd., Zunyi 563000, China; pzpmlhg2004@126.com; 5 School of Materials and Energy, Guangdong University of Technology, Guangzhou 510006, China; 6 Guangdong Province Key Lab of Display Material and Technology, Sun Yat-sen University, Guangzhou 510275, China

**Keywords:** inkjet printing, nanoparticle, metal-organic decomposition, silver thin film, adhesion strength, electrical resistivity

## Abstract

Currently, inkjet printing conductive films have attracted more and more attention in the field of electronic device. Here, the inkjet-printed silver thin films based on nanoparticles (NP) ink and metal-organic decomposition (MOD) ink were cured by the UV curing method and heat curing method. We not only compared the electrical resistivity and adhesion strength of two types of silver films, but also studied the effect of different curing methods on silver films. The silver films based on NP ink had good adhesion strength with a lowest electrical resistivity of 3.7 × 10^−8^ Ω·m. However, the silver film based on MOD ink had terrible adhesion strength with a lowest electrical resistivity of 2 × 10^−8^ Ω·m. Furthermore, we found a simple way to improve the terrible adhesion strength of silver films based on MOD ink and tried to figure out the mechanisms. This work offers a further understanding of the different performances of two types of silver films with different curing methods.

## 1. Introduction

Inkjet printing has been used to print documents in offices for about 50 years. Now, people are aware of its advantages in the electronic field, such as direct patterning without masks, micrometer resolution, low cost, non-contacting printing, etc. [1,2]. Therefore, more and more attentions are paid to the applications of inkjet printing conductive films in electronic products, such as radio frequency identification (RFID) tag [3], thin film transistor [4,5,6], solar cell [7], OLED [8,9], etc. The performance of conductive films mainly relies on the composition of materials and post treatments. Currently, conductive materials widely used in inkjet printing conductive films are silver based inks because silver is more chemically stable than copper and cheaper than gold. In addition, the melting temperature of silver nanoparticles is much lower than bulk silver and decreases as the size of particles decreases, which is beneficial to the low-temperature manufacture [10,11]. So far, silver-based inks mainly include nanoparticles (NP) silver ink and metal-organic decomposition (MOD) silver ink. The NP silver ink consists of silver nanoparticles, organic solvents, dispersants, organic coating surrounding silver particles, and other organic additives, while the MOD silver ink is mainly composed of silver-based precursors and organic solvents [12]. For the NP silver ink, Alessandro et al. [13] realized a power amplifier on a rigid alumina substrate by adopting a hybrid Ag-based ink, whose electrical properties were comparable to that of pure-metallic ink systems. Dearden et al. [14] reported a low curing temperature silver ink for inkjet printing silver films. Zhao et al. [15] improved the electrical conductivity of printed silver films by using a novel carbon nanotubes/silver NP ink. For the MOD silver ink, Nie et al. [16] printed the silver conductive film based on a silver citrate conductive ink. Kalio et al. [17] developed a lead-free silver ink for the front side metallization of silicon solar cells. In a word, most previous works [18,19,20,21] only focused on the effects of heat curing on a single silver ink or printing silver films. However, there are few reports on comparing different types of silver inks. Therefore, it is necessary to figure out different features of NP silver ink and MOD silver ink, which can help researchers find their different scope of applications. The posttreatment methods used in conductive films mainly include heat curing, laser sintering [22], electric sintering [23,24], microwave sintering [3], etc. As a low temperature and fast curing method, UV curing was promising in flexible electronics. Therefore, it was also meaningful to study the effects of UV curing on the NP and MOD silver inks.

In this work, NP silver ink and MOD silver ink were chosen to prepare the inkjet-printed silver thin films on glass substrates. UV curing and the traditional heat curing method were used to cure silver films to investigate the effect of different curing methods on silver films. We investigated the effects of two different curing methods on electrical resistivity and adhesion strength of two types of silver films. Furthermore, we also tried to figure out the different mechanisms that two types of silver films were bonded to the glass substrate under different curing methods. 

## 2. Materials and Methods

In our work, the conventional alkali-free glass with a thickness of 0.7 mm was used as the substrates. To remove dust and contamination on the surface, the substrates were ultrasonicated in isopropyl alcohol, tetrahydrofuran, deionized water, and isopropyl alcohol in sequence. Then, the clean substrates were dried for use without an additional pretreatment. The NP silver ink used in inkjet printing was DGP-40LT-15C (purchased from Advanced Nano Products Co. Ltd., Sejong-si, Korea), while the MOD silver ink was TEC-IJ-010 (purchased from Inktec Co. Ltd., Ansan, Japan). A dimatix material printer (DMP-2800) with a 10 pL cartridge was used to print silver films. During the printing, the inks were printed onto the substrates with a drop spacing of 35 μm. In addition, the substrate temperature of the printer was set at 30 and 50 °C in order to get continous silver films based on NP ink and MOD ink, respectively. After the printing, the silver films were cured by UV curing or heat curing on a hot plate under ambient conditions. The UV light curing system used in UV curing was IntelliRay UV0832 (Uvitron International Inc, West Springfield, MA, USA) and the power of UV lamp in the system is 600 W.

The electrical resistivity of the films was calculated from the equation of $\rho =R_{S}\times h$, where ρ is the electrical resistivity, R_s_ is the sheet resistance, and h is the thickness of films. The sheet resistance and thickness were measured by a digital four-probe tester (KDY-1, Guangzhou Kunde Co. Ltd., Guangzhou, China) and a step profiler (Dektak, Veeco, Plainview, NY, USA), respectively. A scanning electron microscopy (SEM, NOVA NANOSEM 430, FEI, Hillsboro, OR, USA) with an energy dispersive X-ray spectrometer (EDS) was used to obtain a relative element content, surface information, and interface information between silver films and substrates. In addition, the adhesion strength of silver films was tested by the tape test in which we printed 64 square silver films with an edge length of 0.7 mm onto the substrates and used the 3M tape to test the adhesion strength.

## 3. Results and Discussion

**Figure 1:**
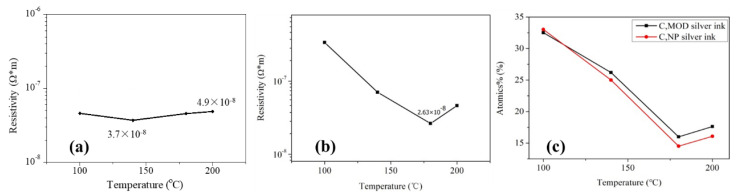
Electrical resistivity and relative carbon content of silver film at different heat-cured temperatures: (**a**) Silver films based on the nanoparticles (NP) ink were heat cured for 30 min; (**b**) silver films based on the metal-organic decomposition (MOD) ink were heat cured for 10 min; (**c**) relative carbon content of heat-cured silver films.

**Figure 2:**
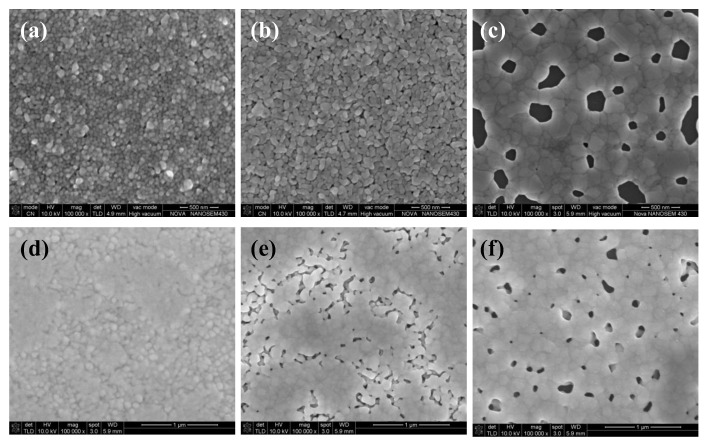
SEM images of heat-cured silver films: Silver films based on the NP ink were cured in (**a**) 100, (**b**) 140, and (**c**) 200 °C for 30 min, respectively; silver films based on the MOD ink were cured in (**d**) 100, (**e**) 140, and (**f**) 200 °C for 10 min, respectively.

Figure 1a,b shows the influence of annealing temperature on the electrical resistivity of two types of silver films, in which the sample in 25 °C was dried naturally without additional curing and just prepared for comparison (not shown). A digital four-probe tester was used to measure sheet resistance and the electrical resistivity of the films was calculated from the equation of $\rho =R_{S}\times h$. The silver film in 25 °C was not conductive (not shown), indicating that a large number of organic coatings existed in the silver films. As shown in Figure 1a,b, the lowest electrical resistivity of the silver films based on NP ink (3.7 × 10^−8^ Ω·m) was larger than that of the silver films based on MOD ink (2.6 × 10^−8^ Ω·m). The electrical resistivity of two types of silver films both decreased dramatically with the increasing temperature at first because of the evaporation of solvent which was consistent with the decreasing relative carbon content, as shown in Figure 1c. However, when the temperature continued to increase, the electrical resistivity increased slightly. Compared with Figure 2a,c or Figure 2d,f, there were more pores with a large size on the surface of silver films in 200 °C. It indicated that the faster evaporation rate caused more pores in silver films, and a faster curing rate made the silver inks solidify more quickly so that less pores could be eliminated by the flow of liquid inks, which caused a worse mutual contact between the silver particles. On the other hand, the rapid solidification of silver ink resulted in a small amount of organic substances trapped inside silver films, which was consistent with a slightly increasing relative carbon content in 200 °C (Figure 1c). As a result, the electrical resistivity of silver films increased. In addition, we speculated that more pores with a large size existing on the surface of silver film based on NP silver ink than that based on MOD silver ink was due to the removal of organic coatings in 200 °C. 

The temperature of the lowest electrical resistivity of the silver films based on NP ink was smaller than that based on MOD ink. It may be ascribed to the existence of the organic coating surrounding the silver particles in the NP ink. The organic coating could not only prevent silver nanoparticles from agglomeration when the silver ink was not cured, but also serve as the binder which leads to a good mutual contact between the silver particles. Therefore, when the solvent was evaporated, organic coating made the silver particle connected with each other so that the temperature (140 °C) of the lowest electrical resistivity of the silver films based on NP ink was lower than that (180 °C) based on MOD ink. However, the existence of organic coating made the lowest electrical resistivity of silver films based on the NP ink larger than that based on the MOD ink. What is more, when organic coating was removed in high temperature, it caused extra pores in the silver films. This explained the phenomenon that the electrical resistivity of the silver films based on NP ink increased slightly in high temperature (Figure 1a). As shown in Figure 2c,f, larger pores were observed in the silver films based on NP ink in 200 °C than that based on MOD ink, which can be ascribed to the removal of organic coating. Compared with NP ink, the silver particles from the MOD ink were not surrounded by organic coating so that higher temperatures were needed to melt silver particles to form the mutual connection between adjacent particles for a lower electrical resistivity. Therefore, less curing time was needed to remove the organic residues in silver films based on MOD ink, which explained why the curing time of the silver films based on MOD ink was 10 min instead of 30 min. As shown in Figure 2e, a large amount of silver particles in silver films based on MOD ink were not connected to the adjacent particles, while the connection between silver particles in silver films based on NP ink in 140 °C was closer (Figure 2b). Therefore, it was necessary to increase the curing temperature to attain the lower electrical resistivity for silver films based on MOD ink.

**Figure 3:**
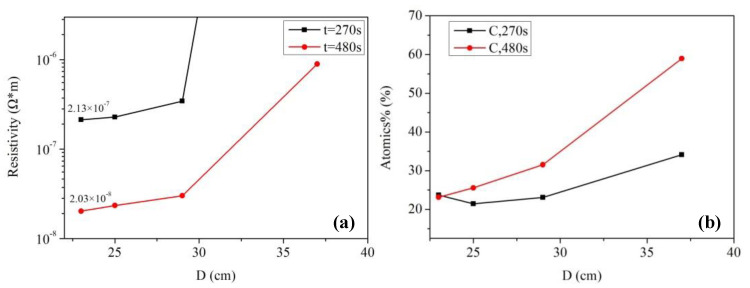
Electrical resistivity and carbon content of ultra violet (UV)-cured silver films based on MOD ink: (**a**) The electrical resistivity of silver films; (**b**) relative carbon content of silver films. D was defined as the distance between the samples and UV lamp.

**Figure 4:**
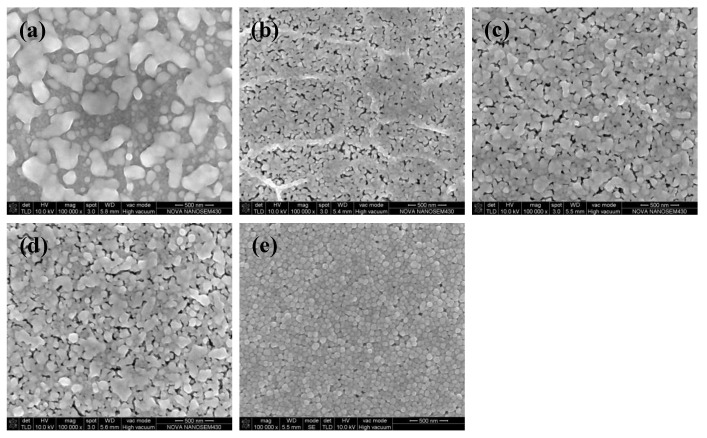
SEM images of silver films with UV curing at: (**a**) D = 37 cm for 270 s; (**b**) D = 23 cm for 270 s; (**c**) D = 23 cm for 480 s; (**d**) D = 25 cm for 480 s; (**e**) D = 25 cm for 480 s [20]. (**a**–**d**) were the films based on MOD ink while (**e**) was the film based on NP ink.

Figure 3a shows the electrical resistivity of silver films based on MOD ink with different UV curing conditions. The electrical resistivity of silver films decreased with the decreasing D (distance between UV lamp and silver films), which was consistent with the decreasing carbon content shown in Figure 3b. In addition, the electrical resistivity decreased with the increase of curing time. The silver film with a low electrical resistivity of ~2.03 × 10^−8^ Ω·m was obtained at D = 23 cm for 480 s by UV curing. Furthermore, the electrical resistivity (~2.03 × 10^−8^ Ω·m) of silver films with UV curing was smaller than the heat-cured one (2.63 × 10^−8^ Ω·m, in Figure 1b), which can be ascribed to the low temperature and good thermal uniformity for the UV curving method. Since less pores were formed in low temperature, the lowest electrical resistivity of silver films with UV curing was lower than the heat-cured one. However, the silver film with UV curing at D = 37 cm for 270 s was not conductive in Figure 3a. As shown in Figure 4a, the silver film with UV curing at D = 37 cm for 270 s was discontinuous, leading to the inferior conductivity. As D decreased and the curing time increased, the silver particles in silver films started to melt and merge into bigger particles as shown in Figure 4b,c. As a result, the electrical resistivity of silver films became smaller. As shown in Table 1, the resistivity value (2.03 × 10^−8^ Ω·m) in our work is lower than that in the previous work, and the UV curing shows a great potential in the field of flexible electronics which need a low temperature process.

Our previous work [25] had studied the effects of UV curing on the electrical resistivity of silver films based on NP ink, and the silver film with a low electrical resistivity of ~6.7 × 10^−8^ Ω·m was obtained at D = 25 cm for 480 s. The electrical resistivity of the silver film based on NP ink was about 2.5 × 10^−7^ Ω·m when it was cured by UV at D = 37 cm for 180 s. By contrast, when D = 37 cm, the silver film based on MOD ink was not conductive until the UV curing time was increased from 270 to 480 s, as shown in Figure 3a. For the precursor in MOD, the process of decomposing and forming silver particles took a lot of energy (UV irradiation: 600 W, D = 23 cm for 480 s), so it needed plenty of time (~480 s) to form the conductive film when D was large. However, the electrical resistivity of the silver film based on MOD ink was much smaller than that of the silver film based on NP ink when they were UV cured at D = 25 cm for 480 s. The relatively large resistivity of the silver film based on NP ink can be ascribed to the organic coating surrounding silver particles. Since the organic coating could prevent silver particles from merging into bigger particles, and UV curing could not remove it completely because of the relatively low energy. As shown in Figure 4e, the silver particles were closely packed but they did not melt and merge into bigger particles. However, the silver particles from the MOD silver ink were not surrounded by organic coating, so they were able to melt and merge into big particles in low temperature (Figure 4d). Therefore, the lowest electrical resistivity of the silver film based on MOD ink (~2.03 × 10^−8^ Ω·m) was much lower than that based on NP ink when they were UV cured at D = 25 cm for 480 s.

**Figure 5:**
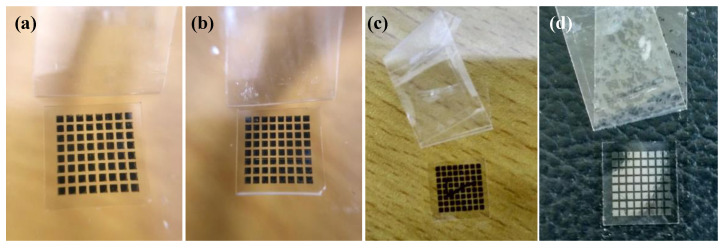
Photo of silver films based on NP ink after the tape test: (**a**) Heat cured in 80 °C for 30 min; (**b**) heat cured in 140 °C for 30 min; (**c**) UV cured at D = 37 cm for 180 s; (**d**) UV cured at D = 25 cm for 480 s.

**Figure 6:**
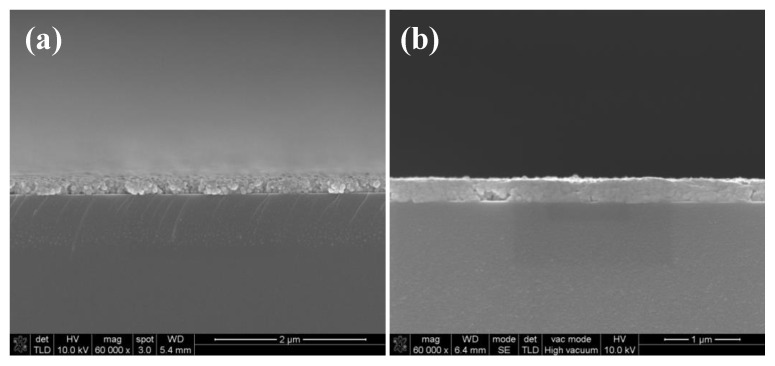
SEM images of the cross section of silver films based on NP ink: (**a**) Heat cured in 140 °C for 30 min; (**b**) UV cured at D = 25 cm for 480 s.

**Figure 7:**
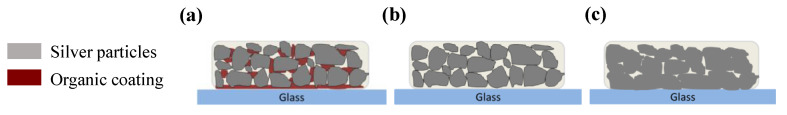
Schematic of the possible adhesion mechanism of (**a**) silver films based on NP ink; (**b**) silver films based on MOD ink cured in low temperature; (**c**) silver films based on MOD ink cured in high temperature.

The adhesion strength of silver films on the glass substrate were characterized by a tape test according to the ASTM international standard [26]. The test steps are as follows: First, an 8 × 8 silver electrodes array with an edge length of 0.7 mm was inkjet printed on the glass substrate and cured in succession. Then, we put the tape on the surface of the silver electrodes and extruded the bubbles in the tape. After resting in 90 s, we tore the tape at a 180° angle from the substrate and observed the falling off of the silver electrodes. Figure 5 shows the adhesion strength of silver films based on NP ink. We found that silver films based on NP ink had good adhesion strength on the glass substrate and almost all of the square films were not detached from the substrates. The adhesion strength of silver electrodes on the glass substrate was about 4–5 B according to the ASTM international standard [26]. We speculated that the remaining organic coating surrounding the silver particles may bond a silver film with the substrate. To figure out the mechanism, we observed SEM images of the cross section of silver films shown in Figure 6. Since the organic coating in NP silver ink usually has a relatively high boiling point and will not be removed in low temperature, the remaining organic coating may serve as the binder [27] and the interfacial pores can be filled with the coating, improving the adhesion strength of silver films, as shown in Figure 7a. As shown in Figure 6, silver particles in the interface between the substrates and films cured by two curing methods had a really close contact with the substrates. What is more, compared with the silver film cured in 140 °C (Figure 2b,e), there were no apparent edges of particles inside the UV-cured silver film and few pores in the interface. It indicated that the silver particles were surrounded by organic coating in low temperature. Therefore, the organic coating bonded silver film with the substrates, leading to the good adhesion strength of silver films is based on NP ink.

**Figure 8:**
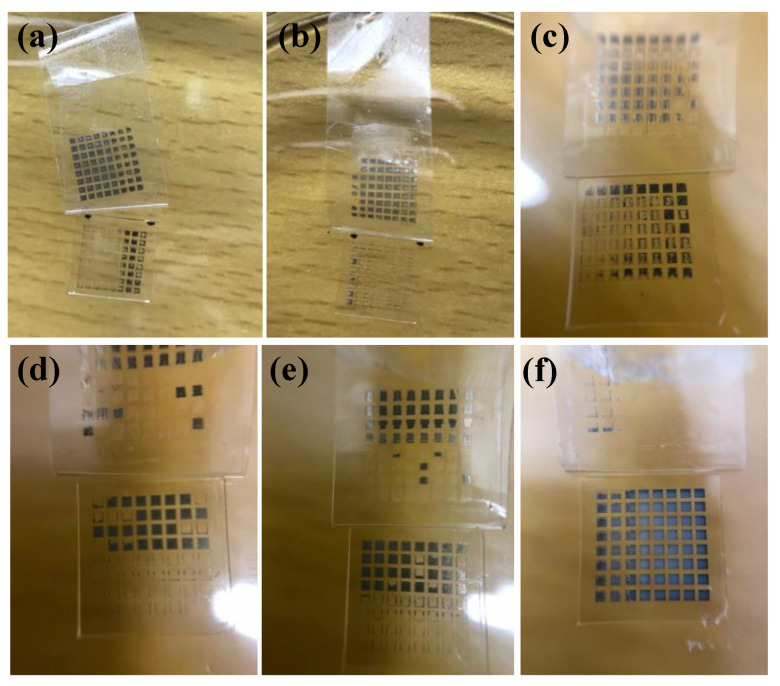
Photos of silver films based on MOD ink after the tape test: (**a**) UV cured at D = 37 cm for 480 s; (**b**) UV cured at D = 29 cm for 480 s; (**c**) UV cured at D = 23 cm for 480 s; (**d**) heat cured at 160 °C for 10 min; (**e**) heat cured at 180 °C for 10 min; (**f**) heat cured at 200 °C for 10 min.

Without the existence of organic coating, the adhesion strength of silver films based on MOD ink was supposed to be worse than that based on NP ink. Figure 8 shows the adhesion strength of silver films based on MOD ink. When silver films were cured by UV curing or heated at 180 °C, the adhesion strength was poor and most of the square films were detached from the substrates, which was consistent with our assumption. Since the solvent could be removed in a really low temperature and there was no organic coating in the MOD silver ink, the connection between silver films and substrates was poor. However, when silver films were heat cured, the adhesion strength increased with the increasing temperature. When the silver film was heat cured at 200 °C for 10 min, few square films were detached from the substrate, indicating that the mechanism of connecting silver films based on MOD ink with the substrates was probably different from that based on NP ink. 

**Figure 9:**
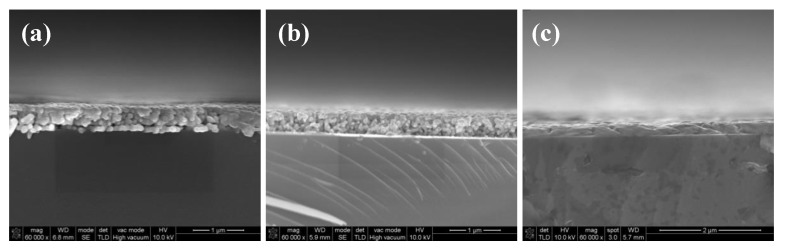
SEM images of cross section of heat-cured silver films based on MOD ink at: (**a**) 160 °C; (**b**) 180 °C; (**c**) 200 °C for 10 min.

As shown in Figure 9a, many pores existed in the interface and most of the silver particles were not melted and merged into big particles when the film was cured in 160 °C. As the temperature increased to 180 °C, the size of the pores in the interface became smaller due to the melting of the particles shown in Figure 9b. Without the existence of the organic coating, the pores in the interface of the silver films based on MOD ink led to a poor connection between the films and substrates, as shown in Figure 7b. Consequently, the adhesion strength of the silver films cured in relatively low temperature was relatively poor. As shown in Figure 9c, when the film was cured at 200 °C, all silver particles were melted and no apparent pores were found in the interface, which indicated that the contacting area between the silver film and the substrate became larger and the connection between the film and the substrate became better. Therefore, the amount of the pores in the interface can be reduced by melting the particles in high temperature and a few pores meant a good connection between the films and substrates, leading to the good adhesion strength [28,29]. In addition, we speculated that a higher temperature could probably promote the mutual diffusion between silver films and substrates [30], which was needed to be proved in the future work.

**Figure 10:**
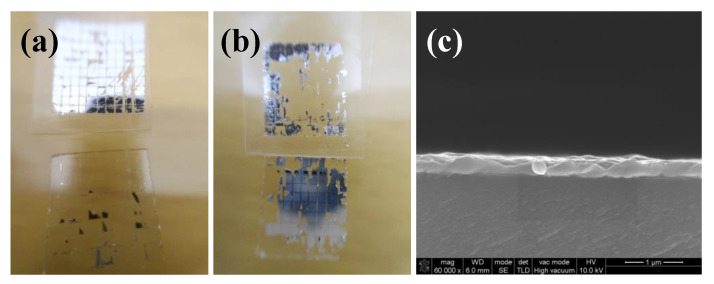
Photos of silver films deposited by DC magnetron sputtering after the tape test: (**a**) Heat cured at 100 °C for 10 min; (**b**) heat cured at 200 °C for 10 min; (**c**) SEM image of cross section of silver films heat cured at 200 °C for 10 min.

To verify our hypothesis, the silver films prepared by DC magnetron sputtering were used for comparison to eliminate the interference of the residue organic substance. The silver films were cured on hot plate at a temperature of 100 and 200 °C for 10 min, respectively. According to the results of the tape test shown in Figure 10, the adhesion strength of the silver film cured at 100 °C was really poor. However, when the temperature increased to 200 °C, the adhesion strength of the silver film was improved significantly. As shown in Figure 10c, the silver particles were melted and no apparent pores existed in the interface, which was similar to Figure 9c. It indicated that the connection between the silver films and glass substrates could be enhanced by increasing the curing temperature without the help of organic coating. Correspondingly, the adhesion strength increased with the increase of curing temperature. By comparing Figure 8f with Figure 10b, the adhesion strength of the silver film fabricated by DC magnetron sputtering was still worse than that of the inkjet-printed silver film based on MOD ink, indicating that there were possibly some residual organic substances on the interface between the substrate and silver film based on MOD ink. Therefore, the adhesion strength of silver film based on MOD ink was mainly affected by the curing temperature (or pores in the interface) and residual organics.

## 4. Conclusions

In conclusion, the NP silver ink and MOD silver ink were used to prepare silver films which were cured by UV curing or the heat curing method, and the effects of two curing methods on the electrical resistivity and adhesion strength of two types of silver films were systematically investigated. The silver films based on NP ink had worse conductivity than that based on MOD ink due to the existence of organic coating in NP ink. In our work, for silver films based on NP ink, the lowest electrical resistivity of the UV-cured films was almost two times larger than that of the heat-cured films because the organic coating stopped silver particles from merging into big particles in low temperature. For silver films based on MOD ink, the lowest electrical resistivity of the UV-cured films (~2.03 × 10^−8^ Ω·m) was similar to that of the heat-cured films (2.6 × 10^−8^ Ω·m), which means that silver films based on MOD ink could attain a low electrical resistivity in the low-temperature process. However, silver films based on MOD ink had to be cured in a relatively high temperature to attain good adhesion strength while the silver films based on NP ink could attain good adhesion strength in a relatively low temperature with the help of organic coating. Furthermore, the existence of the remaining organic coating may cause interface problems of multilayers’ structure. These results exhibited that our work could offer a further understanding of the different features of inkjet-printed silver thin films based on NP ink and MOD ink and help other researchers choose proper conductive materials and curing methods to fabricate different electronic devices. Next, we plan to study the flexible electronic devices based on printing silver electrodes in the future.

## Figures and Tables

**Table 1:** Comparison of resistance of silver films between the previous work and this work.

Ref.	Ink Type	Preparation	Post Treatment	Electrical Resistivity/(Ω·m)
2	MOD	Inkjet printing	heat curing (170 °C)	8.4 × 10^−8^
13	NPs	Inkjet printing	heat curing (150 °C)	(3–4) × 10^−7^
14	NPs	Inkjet printing	heat curing (150 °C)	1.8 × 10^−7^
20	NPs	Inkjet printing	UV curing	6.69 × 10^−8^
This work	MOD	Inkjet printing	UV curing	2.03 × 10^−8^
